# A Systematic Review Examining the Relationship Between Habit and Physical Activity Behavior in Longitudinal Studies

**DOI:** 10.3389/fpsyg.2021.626750

**Published:** 2021-03-04

**Authors:** Katharina Feil, Sarah Allion, Susanne Weyland, Darko Jekauc

**Affiliations:** Department of Health Education and Sport Psychology, Institute of Sports and Sports Science, Karlsruhe Institute of Technology, Karlsruhe, Germany

**Keywords:** habit, physical activity, longitudinal, maintenance, review, automaticity

## Abstract

**Purpose:** To explain physical activity behavior, social-cognitive theories were most commonly used in the past. Besides conscious processes, the approach of dual processes additionally incorporates non-conscious regulatory processes into physical activity behavior theories. Habits are one of various non-conscious variables that can influence behavior and thus play an important role in terms of behavior change. The aim of this review was to examine the relationship between habit strength and physical activity behavior in longitudinal studies.

**Methods:** According to the PRISMA guidelines, a systematic search was conducted in three databases. Only peer-reviewed articles using a longitudinal study design were included. Both, habit and physical activity were measured at least once, and habit was related to physical activity behavior. Study quality was evaluated by assessment tools of the NHLBI.

**Results:** Of 3.382 identified publications between 2016 and 2019, fifteen studies with different study designs were included. Most studies supported that positive correlations between habit and physical activity exist. Some positive direct and indirect effects of habit on physical activity were detected and only a minority of studies showed the influence of physical activity on habit strength. Studies differentiating between instigation and execution habit found positive correlations and revealed instigation habit as a stronger predictor of physical activity. The quality of studies was rated as reasonable using assessment tools of the NHLBI.

**Conclusion:** This review revealed a bidirectional relationship between habit and physical activity. Whether habit predicts physical activity or vice versa is still unclear. The observation of habit influencing physical activity may be most appropriate in studies fostering physical activity maintenance while the influence of physical activity on habit may be reasonable in experimental studies with physical activity as intervention content to form a habit. Future investigations should differentiate between habit formation and physical activity maintenance studies depending on the research objective. Long-term study designs addressing the complexity of habitual behavior would be beneficial for establishing cue-behavior associations for the formation of habits. Furthermore, studies should differentiate between instigation and execution habit in order to investigate the influence of both variables on physical activity behavior independently.

## Introduction

Regular physical activity reduces the risk of premature mortality (Warburton and Bredin, 2017) and has a preventive effect on chronic diseases, such as coronary heart disease and diabetes mellitus (Reiner et al., 2013; Musich et al., 2017). Besides the physiological health benefits of physical activity, positive associations between physical activity and mental health were also found (White R. L. et al., 2017). However, the physical activity level of adults does not meet the international physical activity guidelines of the World Health Organization (WHO, 2010). A worldwide study found that 31% of adults are physically inactive with rates up to 43% in Western countries like America and east Mediterranean regions (Hallal et al., 2012).

In the past, physical activity behavior was most commonly explained by social-cognitive theories, such as the Theory of Planned Behavior (TPB) (Ajzen, 1991) or the Health Action Process Approach (HAPA) (Schwarzer, 2008). The intention towards the behavior is a fundamental component of these theories. Nevertheless, there remains an underestimated gap between intention and behavior. Constructs such as self-efficacy beliefs (Bandura, 2004) or action planning (Schwarzer, 2008) aim to bridge this intention-behavior gap, but evidence shows that only a small amount of physical activity behavior can be predicted by explicit processes (McEachan et al., 2011; Rhodes and Dickau, 2012).

Because conscious processes do not satisfactorily lead to health behavior changes, scientists suggest incorporating implicit regulatory processes into physical activity behavior theories (Marteau et al., 2012; Rhodes and Dickau, 2012; Sheeran et al., 2013; Strobach et al., 2020). A dual-process approach differentiates two processes as a basis for decision making toward a behavior (Evans and Stanovich, 2013). Type 1 is characterized as fast, nonconscious, and automatic, while type 2 is described as slow, conscious, and controlled. So far, physical activity behavior preceding from type 2 has mainly been the subject of investigations, but more recent studies referring to non-conscious processes have increasingly gained attention.

Habits are one of various non-conscious processes that can influence behavior (Lally and Gardner, 2013; Gardner, 2015; Rebar et al., 2016; Gardner and Lally, 2018). Gardner and Lally (2018, p. 209) defined habit as “a process whereby encountering a cue triggers an impulse to perform an action that has, through learning, become a learned response to the cue.” Health behavior can become habitual, but authors argue that habit and behavior are not the same (Maddux, 1997). A framework was introduced by Gardner and Lally (2018) based on their previous work (Lally and Gardner, 2013) for understanding the habit formation process. According to their model, an intentional decision toward the behavior (stage 1) and a subsequent action initiation (stage 2) is necessary in order to show a behavior for the first time. Habit formation eventuates from repeating this behavior (stage 3a) and developing cue-behavior associations (stage 3b). Behavioral repetition is a central part of habit formation. Thus, as the habit forms, it influences subsequent behavior. As Gardner (2015) pointed out, the relationship between habit and behavior might be bidirectional and evolves over time. That behavior determines habit and habit determines behavior is crucial to understand the habit literature.

Complex health behaviors such as physical activity can be understood as a sequence of simple habitual actions (Maddux, 1997). Therefore, literature suggests a distinction between habitual initiation and habitual performance of the behavior. The decision toward physical activity behavior through unconsciously triggered impulses is called instigation habit whereas the execution habit is defined as habitually doing physical activity (Gardner, 2015; Gardner et al., 2020a). The performance of physical activity can become habitual through automatically executed sub-actions. While deciding to exercise builds the instigation habit (“Deciding to exercise… [… is something I do automatically]”), sub-actions like going to the gym or performing the exercise tasks at the gym are called execution habit (“Once I am exercising… [… is something I do automatically]”) (Phillips and Gardner, 2016; Gardner et al., 2020a). In a current debate several authors critically discussed the definitions and relevance of instigation and execution habit toward physical activity behavior (Hagger, 2019, 2020; Gardner et al., 2020a). However, this discussion is still ongoing due to a lack of experimental studies focusing on these different constructs. Meanwhile, other scientists such as Kaushal et al. (2017a) extended the approach of instigation and execution habit, dividing exercise into two behavioral phases: preparatory and performance phase. The preparatory phase involves all behaviors until an individual reaches an exercise-ready state (“When I prepare to exercise… […is something I do automatically]”). The performance phase starts when exercising begins (“When I exercise… […is something I do automatically]”) (Kaushal et al., 2017a). Kaushal et al. (2017a) assumed that the habituation of the preparation phase in physical activity behavior could be a more promising approach than focusing on the habituation of the performance phase because preparatory actions may be less complicated and shorter in duration than exercising itself.

Previous studies revealed that habit formation processes can affect health behaviors positively. In their meta-analysis, Gardner et al. (2011) found 23 habit-behavior relations across 21 data sets presenting a moderate-to-strong correlation between habit and physical activity behavior (fixed *r* = 0.43, random *r* = 0.44, *p* < 0.001). A recent systematic review by Rebar et al. (2016) supports these findings. Habit was positively associated with physical activity (d = 0.67) which was confirmed in 13 out of 15 included studies.

Further, the role of habit as a moderator between intention and health behavior was investigated (Gardner, 2015; Rebar et al., 2016). A narrative review by Gardner (2015) showed that the predictive effect of intention on health behavior weakens when habit strength increases. Eleven included physical activity studies revealed mixed findings, but seven studies supported that an increasing habit debilitates the intention-behavior relationship. Rebar et al. (2016) confirmed these findings and detected in most studies that intention-behavior associations are stronger for participants with lower habit strength compared to those with higher. However, Rebar et al. (2019) critically discussed in a current paper that this assumption is misleading due to linear modeling analysis. The authors observed the interrelatedness and typical asymmetrical distributions of intention and behavior using a simulated data set. They revealed similar moderating effects of high as well as low habits on the intention-behavior relationship. The previously moderating effects of habit may be based on statistical by-products of inappropriate model analyses. Therefore, Rebar et al. (2019) argued that strong conclusions about the moderating effect of habit should be reconsidered due to a high risk of misinterpreting tests. Additional to suitable moderation testing, intention-behavior profiles based on decisional intentions and subsequent behavior are recommended to enhance validity of findings (Rebar et al., 2019). Altogether, the moderating effect of habit is still unclear and the direct effect of habit on physical activity behavior has not been sufficiently investigated yet.

A small amount of observational and intervention studies in the narrative review by Gardner (2015) analyzed whether health behavior predicted habit strength. Here, estimated effect sizes must be interpreted with caution, because most studies tried to predict ongoing habits. To examine the effect of physical activity on habit, habit formation studies, where new habits emerge, would be more suitable (Gardner, 2015).

Current literature suggests that building an instigation habit is more valuable for the maintenance of behavior than an execution habit (Gardner and Lally, 2018). Instigation habit can be understood as “the direct activation of action” (Phillips and Gardner, 2016, p. 70) and therefore automatically generates the behavior performance (Phillips and Gardner, 2016). Thus, researchers assume behavior frequency is being regulated by instigation rather than execution habit (Gardner and Lally, 2018).

Further research with longer-term outcome measures will be essential so that the impact of non-conscious processes and health behavior maintenance can be observed (Rebar et al., 2016). Gardner (2015) recommended longitudinal study designs and within-person analyses to test causal influences on habit strength. The formation of habit within each participant should be included in statistical analyses, because between-subject effects only reflect the speed or peak of a groups' habit. Additionally, study quality would increase through multiple measurement occasions over a long time period with follow-up measures (Gardner, 2015; Rebar et al., 2016; Gardner and Lally, 2018).

The latest systematic review on habits and physical activity was conducted by Rebar et al. (2016) and revealed that implicit processes, such as habit, partially determine physical activity behavior. Recommendations for future studies were made and, therefore, we expect the latest studies to apply these guidelines. Studies published in the same time period might not have benefited from the results of the review by Rebar et al. (2016), but the claim for longitudinal study designs had already been made by Gardner et al. (2011). Consequently, the current review searched for longitudinal studies since 2016 to summarize the latest evidence in this field of research. The superior objective is to deepen the knowledge regarding the relationship between habit and physical activity behavior. Thus, the aim of this review was to examine the relationship between habit formation or habit strength and the acute level of physical activity behavior in longitudinal studies. Assuming that relevant studies conducted correlational analyses, our first hypothesis is formulated independently of the study design. Therefore, we expected (1) positive correlations between the variables habit and physical activity. Particularly, we hypothesized that (2a) higher habit strength leads to an increased level of physical activity and that (2b) an increased level of physical activity leads to higher habit strength. Based on the latest research in this field we assumed that (3) the relationship between instigation habit and physical activity is stronger than the relationship between execution habit and physical activity.

## Methods

This systematic review was conducted according to the PRISMA guidelines (Liberati et al., 2009).

### Eligibility Criteria

Studies meeting the following inclusion criteria were included in this review: (1) a measurement of physical activity with physical activity defined “as any bodily movement produced by skeletal muscles that results in energy expenditure” (WHO, 2020). Here, we also include intervention studies when the content of the intervention was clearly physical activity, although there was no statistical analysis testing the relationship between a concrete measurement of physical activity and habit. (2) A measurement of habit referring to physical activity specified as the self-reported habit index (SRHI) or the self-reported behavioral automaticity index (SRBAI) or a short version of these questionnaires. (3) Longitudinal study design with at least two measurement occasions. (4) Only published papers in peer-reviewed journals between April 2016 and November 2019 written in English or German. Exclusion criteria regarding the characteristics of the study population were not declared.

### Search

The search was conducted on November 15, 2019, in the databases Web of Science, Pubmed, and Scopus. The constitution of the search term was based on the systematic review by Rebar et al. (2016), but was slightly adapted due to the focus on habit only and not on automatic associations or priming effects. Furthermore, we added a third part to the term to ensure a longitudinal study design. Our search terms consisted of the following three types of related terms:

1. Physical activity related terms: “physical activity” OR “exercise^*^” OR “sedentary” OR “walking”

2. Habit related terms: “SRHI” OR “habit” OR “habits” OR “habitual” OR “implicit”

3. Study design: “longitudinal” OR “maintenance” OR “within-subject” OR “with-in-person” OR “intervention”

At least one term within these three types had to be met in order to be included in the study. Furthermore, we screened reference lists and citations of eligible studies to identify additional relevant studies.

### Study Selection

The search was executed by three independent reviewers with the data management system EndNote X9. In the first selection step, a screening of titles was carried out, followed by an inspection of eligibility criteria in the abstracts. Abstracts meeting the criteria were further examined by reading the full text of articles. Full texts were also read for studies with abstracts providing insufficient information about eligibility. Potential studies for inclusion in the review were scanned by all reviewers. Disagreements regarding inclusion were solved by discussion. Consensus was achieved in 100% of the cases.

### Data Extraction

We extracted the data from full-text articles, related publications, and supplementary material. The data was filled into an extraction form which contains source (authors, year of publication, country of origin), theory/model, study design (theory, measurement points, statistics), sample (setting, sample size, mean age), treatment (length of intervention, treatment, duration, frequency), outcome (measurements of physical activity and habit), and results. Only additional measurements and results related to the relationship between habit and physical activity were reported. Additional outcomes not relevant to the research question were not included in the data extraction.

### Quality Assessment

To assess the risk of bias due to flaws in design and implementation of the studies, quality assessment tools of the National Heart, Lung, and Blood Institute (NHLBI, 2014) were used. According to the study design, tools for controlled intervention studies, observational cohort and cross-sectional studies, and before-after studies with no control group were applied. The study ratings incorporated selection bias across participants, study design, confounders, blinding of researchers and participants, data collection methods, and drop-outs. Quality evaluation occurred similarly for each tool, so that a unitary overall rating was possible, and comparability of the study quality was ensured. The global rating differentiated between poor, fair, and good study quality. Poor ratings indicated a significant risk of bias. Fair studies are susceptible to some bias, thus, these results should be considered as insufficiently valid. Least risk of bias is given in good-rated studies, whose results can be considered as valid. Three reviewers conducted the assessment independently. Discrepancies in the evaluation of items were marginal and immediately resolved by discussion in 100% of the cases.

## Results

### Study Selection

**Figure 1:**
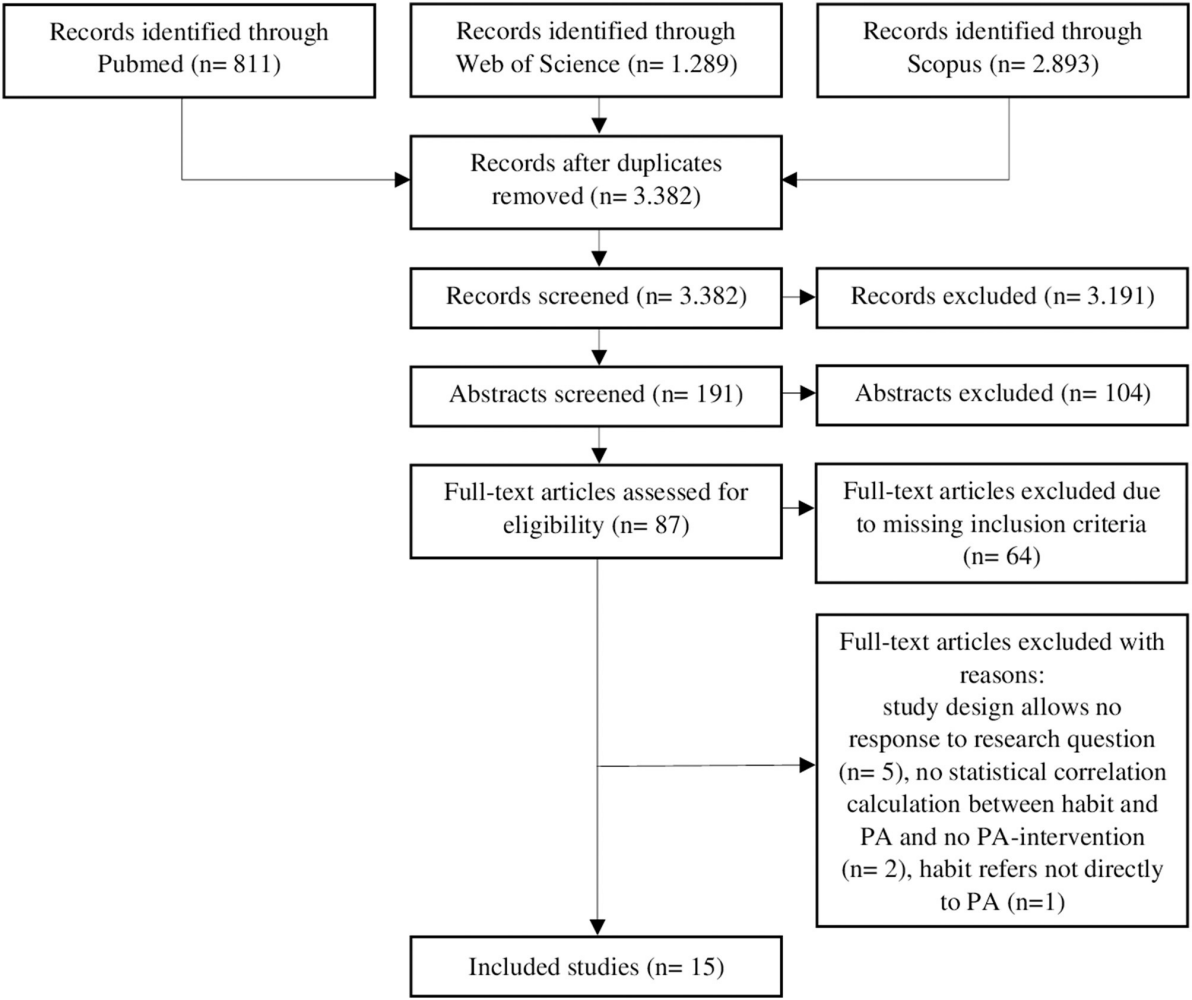
Flow diagram of study selection process. PA: physical activity.

After removing duplicates, a total of 3.382 studies were identified and 191 studies remained after screening titles. Abstract screening revealed 87 eligible full-text articles for full-text screening. During full-text reading, 64 studies were excluded as they did not meet the inclusion criteria. An additional five studies were eliminated because the results did not answer the research question of this review. These included a validity study of another measurement of habit (Boiché et al., 2016), a feasibility study with insufficient statistical results (Ashe et al., 2019), and studies not reporting habit as an individual variable and its relationship to physical activity (Phillips et al., 2016; Duan et al., 2017; Howlett et al., 2019). One study was excluded, because habit did not refer directly to physical activity but to wearing a Fitbit (Ellingson et al., 2019). Furthermore, two studies did not meet the inclusion criterion of connecting habit to physical activity through either a statistical analysis or using physical activity as the content of intervention (Hamilton et al., 2019; Wallmann-Sperlich et al., 2019). In total, 15 studies were included in this systematic review (see Figure 1).

### Study Characteristics

Country, study design, background theories, outcome measurement tools, and statistical analysis of included studies are shown in Table 1.

**Table 1:** Study characteristics.

**Study characteristics**	**Number**
**Country**	
Europe	8
North America	4
Australia	3
**Study design**	
Longitudinal observational design	9
Longitudinal two-arm intervention design	3
RCT	3
**Theories**	
Social-cognitive theories (TPB, HAPA, strength model of self-control)	6
Habit theories	3
Dual-process approach (RIM, dual-process model)	1
No theory	5
**Measurement of habit**	
SRBAI	11
SRHI	2
Items of SRHI and SRBAI	2
Single-item SRHI	2
**Measurement of physical activity**	
Questionnaire	8
Single items	7
Accelerometer	2
Electronic daily diary report	1
**Statistical analysis**	
Correlation	12
Regression	7
Structural equation model	4
Mixed model/ general linear model	2
Mediation analysis	1
ANOVA	1

Target populations in the studies were adults (Schwarzer et al., 2017; Bird et al., 2018), university students and staff (Allom et al., 2016; Phillips and Gardner, 2016; Pfeffer and Strobach, 2018), cardio-vascular patients (Fournier et al., 2018), gym members (Kaushal et al., 2017a, 2018), older adults (Arnautovska et al., 2016; van Bree et al., 2016, 2017; White I. et al., 2017), office employees (Fournier et al., 2016), pregnant women (Mullan et al., 2016), and parents (Rhodes et al., 2019b). The inquiry period ranged from 1 week (Allom et al., 2016; Mullan et al., 2016; Pfeffer and Strobach, 2018; Rhodes et al., 2019b) to 2 years (Bird et al., 2018). The number of post-baseline measurement occasions varied between 1 (Allom et al., 2016; Arnautovska et al., 2016; Phillips and Gardner, 2016; Kaushal et al., 2017a, 2018; Bird et al., 2018; Pfeffer and Strobach, 2018) and 4 years (Fournier et al., 2018). Moreover, weekly measurements were applied only in two studies, one reporting habit (Fournier et al., 2016) and one reporting physical activity (Rhodes et al., 2019b). In intervention studies, the length of treatment differed between eight (Kaushal et al., 2018) to 28 weeks (Fournier et al., 2016). Besides widely known cognitive theories like TPB and dual-process approaches, very few studies named a concrete habit theory like the habit model by Lally and Gardner (2013). Most studies focused on the construct of habit as their theoretical basis and some of them did not refer to an existing theory by name (no theory). In addition to the background theories, four studies described further theoretical approaches to implement the study procedure. In this case, theories were applied to examine the adaptability of the constructs or to facilitate the implementation process of an intervention. These theories were an extended version of the TPB (Bird et al., 2018), a dual-process approach (Kaushal et al., 2017a), and the Multi-Process Action Control (M-PAC) (Kaushal et al., 2018; Rhodes et al., 2019b).

### Quality Assessment

A total of five studies were rated as poor (Allom et al., 2016; Arnautovska et al., 2016; Mullan et al., 2016; Bird et al., 2018; Kaushal et al., 2018), as fair (van Bree et al., 2016, 2017; Kaushal et al., 2017a; Schwarzer et al., 2017; White I. et al., 2017), and as good quality (Fournier et al., 2016, 2018; Phillips and Gardner, 2016; Pfeffer and Strobach, 2018; Rhodes et al., 2019b). When no power analysis was conducted (Allom et al., 2016; Arnautovska et al., 2016; Mullan et al., 2016; Bird et al., 2018) and no repeated measurement of exposure and outcome was operated (Allom et al., 2016; Arnautovska et al., 2016; Mullan et al., 2016), quality was rated as poor. Additionally, in one poor-rated study, blinding was not reported and data of intervention participants who did not attend were used for analysis in the control group (Kaushal et al., 2018). Also, poor quality was assessed for one study using only a single item of the SRHI and in which the timeframe was not appropriate for only two measurement occasions (Bird et al., 2018). Another reason for poor quality was that the outcome measures were not reported as valid and reliable (Mullan et al., 2016). The rating of each item can be found in the Supplementary Material.

### Relationship Between Habit and Physical Activity

#### Hypothesis 1: Positive Correlations Between the Variables Habit and Physical Activity

Nine studies examined correlations between habit and physical activity through bivariate correlation analysis and eight studies confirmed the first hypothesis of a positive correlation (Allom et al., 2016; Arnautovska et al., 2016; Mullan et al., 2016; Phillips and Gardner, 2016; van Bree et al., 2016, 2017; Kaushal et al., 2017a, 2018; Schwarzer et al., 2017; Pfeffer and Strobach, 2018; Rhodes et al., 2019b). The highest positive correlation was revealed by Mullan et al. (2016) (*r* = 0.62, *p* < 0.01) and the lowest by Schwarzer et al. (2017) (*r* = 0.16, *p* < 0.01). Only White I. et al. (2017) reported no significant relation between the two variables; no study reported negative correlations.

#### Hypothesis 2a: Higher Habit Strength Leads to an Increased Level of Physical Activity

The second hypothesis proposes that habit formation leads to an increase in physical activity. Results can be distinguished between direct and indirect prediction of physical activity. Two studies investigated the direct effects of habit on physical activity (Arnautovska et al., 2016; Rhodes et al., 2019b). Arnautovska et al. (2016) revealed that habit influences physical activity directly (β = 0.24, *p* < 0.01) through a structural equation model. Rhodes et al. (2019b) showed that habit was a predictor of physical activity in successful intenders compared to unsuccessful intenders using accelerometry (OR = 1.99, *p* < 0.01) and self-reported measurements (OR = 1.60–2.08, *p* < 0.01–0.05).

Another five studies detected indirect effects of habit on physical activity (Allom et al., 2016; Mullan et al., 2016; Schwarzer et al., 2017; Bird et al., 2018; Pfeffer and Strobach, 2018). Different regression models including psychological and demographic variables were applied to explain physical activity behavior. For example, Pfeffer and Strobach (2018) investigated the association between self-control and physical activity, and assumed that habit moderates or mediates this relationship in university students. The authors conducted this study in a laboratory setting and reported habit as a moderator (ß = 0.23, *p* < 0.01) and mediator (ß = 0.28, *p* = 0.01), predicting physical activity in a model with age, sex, and self-control. Beyond that, the interaction effect between habit and self-control functioned as a moderator toward physical activity (ß = 0.19, *p* = 0.028). Schwarzer et al. (2017) used a digital intervention in European countries to improve physical activity in adults. A structural equation model was applied to examine psychological variables such as motivation, planning, self-monitoring, habit, and physical activity, which were placed in a sequential manner. Influenced by these variables, habit was found as a predictor of physical activity in men (β = 0.17, *p* < 0.01) and women (β = 0.23, *p* < 0.01). Bird et al. (2018) used a sample from a project study focusing on purpose-built infrastructure. They aimed to improve walking and cycling routes in specific regions of the UK. The effect of a high habit on physical activity was analyzed after 1 or 2 years, applying an extended model of the TPB. A high habit was associated with walking and cycling for transport or recreational use. Results show positive effects on walking and cycling for transport with RRR varying between 2.24 (*p* < 0.01) and 3.33 (*p* < 0.05), but inconclusive findings for recreational use. In fact, an increase as well as a decrease in walking for recreation was found. In most cases, the cohort group measured after 2 years yielded a higher effect of habit on physical activity. Allom et al. (2016) looked at the importance of intentions and habits of first generation students transitioning to college and how these life-changes influenced physical activity behavior. Habit was a significant predictor of physical activity (ß = 0.36, *p* < 0.01) in a hierarchical regression analysis. Allom et al. (2016) used the same statistical procedure and revealed similar results (ß = 0.33, *p* < 0.01) in pregnant women.

#### Hypothesis 2b: Increased Level of Physical Activity Leads to Higher Habit Strength

The influence of physical activity on habit strength in two different sample sizes from previous studies was examined in the study of van Bree et al. (2016). Physical activity was used as a mediator between intention and habit. A regression analysis revealed an effect of physical activity on habit in study one (âbcs = 0.18, *p* < 0.01) as well as in study two (âbcs = 0.21, *p* < 0.01). The standardized indirect effect was expressed through âbcs and showed medium to large effects (0.01 = small, 0.09 = medium, 0.25 = large).

Two intervention studies showed that habit significantly increased over time throughout all intervention groups (Fournier et al., 2016, 2018). In Fournier et al. (2016), the first intervention group received a physical activity program and habit promoting cues, while the second group only received a physical activity intervention. Therefore, physical activity might be the central intervention content that leads to a higher level of habit in these cases. Fournier et al. (2016) yielded a higher habit strength in the first group which received additional cues for habit formation (B = 0.05, *p* < 0.01) compared to the second group performing only a physical activity program (B = 0.02, *p* = 0.02). Moreover, the first intervention group reached a significantly higher level of physical activity at follow-up [t_(14)_ = 2.20, *p* = 0.04] than the second intervention group [t_(7)_ = 1.28, *p* = 0.24]. These findings support the assumption that habit formation interventions lead to a higher level of physical activity and would therefore support hypothesis 2a as well. However, in Fournier et al. (2018), subgroups did not differ in habit or physical activity, and physical activity did not significantly change over time.

In another study, van Bree et al. (2017) applied a cross-lagged panel design to investigate whether habit mediates the relationship between prior and later physical activity (physical activity-habit-physical activity) and vice versa (habit-physical activity-habit). Two different sample sizes from previous studies were used to adapt the design. Study 1 revealed a significant mediation effect for the path habit-physical activity-habit (âbcs = 0.01, *p* = 0.006), while the path physical activity-habit- physical activity showed only marginal significance. In study 2, contrary findings were reported with a significant mediation effect for the path physical activity-habit-physical activity (âbcs = 0.03, *p* < 0.01) while the path habit-physical activity-habit was not significant.

#### Hypothesis 3: Relationship Between Instigation Habit and Physical Activity Is Stronger Than the Relationship Between Execution Habit and Physical Activity

Only one study investigated instigation and execution habit separately (Phillips and Gardner, 2016). The authors revealed correlations between instigation habit and physical activity at different occasions (*r*-values between *r* = 0.32–56, *p* < 0.01), whereas lower correlations were found between execution habit and physical activity (*r*-values between *r* = 0.22–0.41, *p* < 0.05 and *p* < 0.01). Multiple regression analysis yielded only instigation habit as a predictor of physical activity at t2 (daily diary report: β = 0.31, t_(3,108)_ = 2.56, *p* = 0.01; physical activity 1 item: β = 0.39, t_(3,107)_ = 3.16, *p* < 0.01). Furthermore, Phillips and Gardner (2016) examined whether the change of physical activity is associated with change in instigation habit. Participants with a high physical activity level at baseline and a decrease over time (high/low group) showed a negative change in instigation habit at t1 (slope = −0.46, *p* = 0.02). The positive change in instigation habit for participants increasing their physical activity level from low to high was not significant (slope = 0.21, *p* = 0.22).

Kaushal et al. (2017a, 2018) focused on preparatory and performance habits of physical activity in gym members as an extended approach to Phillips and Gardner (2016). Both studies revealed correlations between preparatory habit and physical activity and performance habit and physical activity. Preparatory habit showed only marginally higher values (*r* = 0.22–25, *p* < 0.01) compared to performance habit (*r* = 0.20–23, *p* < 0.05 and *p* = 0.01). Structural equation models yielded preparatory habit as a significant predictor for physical activity (ß = 0.20, *p* = 0.03) and change in physical activity (ß = 0.18–20, *p* = 0.04) (Kaushal et al., 2017a). No significant effect of performance habit on physical activity was found (Kaushal et al., 2017a, 2018).

A detailed data extraction of the studies and their results are shown in Table 2.

**Table 2:** Data extraction of included studies.

**References Country of origin**	**Theory/model**	**Study design (measurement occasions, statistics)**	**Sample (final sample size, gender proportion, mean age)**	**Treatment (content, length, duration, frequency)**	**Outcome (measurements of habit and PA)**	**Results**
Allom et al. (2016) Australia	TPB	Longitudinal observational design Baseline (SRHI) follow-up (PA items, 1 week) Bivariate correlation Multiple regression analysis	College students *N* = 101 *M* = 19.6 ± 4.88	No treatment	Habit: SRHI PA: 2 single-items “In the last week, to what extent did you do regular PA?” “How often in the last week did you do regular PA?”	Bivariate correlation between habit and PA (*r* = 0.61, *p* < 0.01) Multiple regression analysis: habit was a sig. predictor of PA (β = 0.36, *p* < 0.01) (model with sex, intention, PBC, intention x habit)
Arnautovska et al. (2016) Australia	TPB, HAPA, RIM, Dual-process model (Presseau et al., 2014)	Longitudinal observational design Baseline (SRBAI) t1 (PA items, 2 weeks) Bivariate correlation, Structural equation model	Older adults *N* = 165 (66.7% female) *M* = 73.8 ± 7.0	No treatment	Habit: SRBAI PA: 3 single-items “On how many days in the past week (past 7 days) have you engaged in at least 30 min of at least moderate-intensity PA?” “In the previous week, how often did you engage in regular PA?” “In the previous week, to what extent did you engage in regular PA?”	Bivariate correlation between habit strength at baseline and PA at t2 (*r* = 0.51, *p* < 0.01) Structural equation model: habit strength sig. predicts PA (ß = 0.24, *p* < 0.01)
Bird et al. (2018) UK	TPB, eTPB	Longitudinal observational cohort design Baseline 2010 (SRHI 1 item, PA self-report) Cohort (1) follow-up in 2011 (SRHI 1 item, PA self-report, 1 year) Cohort (2) follow-up in 2012 (SRHI 1 item, PA self-report, 2 years) Regression analysis	Adults *n* = 1.796 (54.5% female) *n* = 1.465 (56.7% female) No total *M*_age_ (for details see Ogilvie et al. (2011, 2012)	No treatment Data from the iConnect study (Ogilvie et al., 2012) used to examine psychological predictors of change in walking/ cycling for recreation using an extended version of TPB	Habit: SRHI 1 item (walking /cycling for recreation (leisure, health, fitness)/ for transport (to get to places) is something I do automatically without really thinking about it) PA: self-report based on IPAQ (min/week)	Regression analysis (model 3 with PBC, intention, habit, visibility and controlled variables): Associations between high habit and - increase in walking for transport in (2) (*RRR* = 2.24 (1.41–3.57), *p < 0*.01) - increase in walking for recreation in (1) [*RRR* = 1.89 (1.29–2.77), *p* < 0.01] and (2) [*RRR* = 2.36 (1.53–3.65), *p* < 0.01] - decrease in walking for recreation in (1) [*RRR* = 1.67 (1.10–2.53), *p* < 0.05) and (2) [*RRR* = 2.43 (1.52–3.87), *p* < 0.001] - increase in cycling for transport in (1) [*RRR* = 2.89 (1.37–6.06), *p* < 0.01] and (2) [*RRR* = 3.33 (1.31–8.45), *p* < 0.05] Association between habit and increase/decrease in cycling for recreation in (1) and (2) n.s.
Fournier et al. (2016) France	–	Longitudinal two-arm intervention design t1-t28 (SRBAI, weekly) follow-up (IPAQ, 19 months) Mixed model, General linear model	Employees *N* = 39 (57% female*) (1) *n* = 19 (2) *n* = 20 *M* = 47.5 ± 8.29*	28 weeks (1) PA+SMS: PA program two 1-hours sessions per week, text messaging cues before the night before PA sessions and 1-hour before PA sessions (4/week) (2) PA program only	Habit: SRBAI PA: IPAQ	Mixed model: - habit increases over time in both groups, but higher in (1) (*B* = 0.05, *p* < 0.01) than in (2) (*B* = 0.02, *p* = 0.02) General linear model: PA at follow-up higher in (1) *t*_(14)_ = 2.20, *p* = 0.04) than in (2) *t*_(7)_ = 1.28, *p* = 0.24)
Fournier et al. (2018) France	–	Longitudinal two-arm intervention design Baseline (SRBAI, IPAQ) t1 (SRBAI, IPAQ, 5 months) t2 (IPAQ, 7 months) t3 (IPAQ, 9 months) t4 (SRBAI, IPAQ, 12 months) Mixed model, General linear model	Cardio-vascular patients *N* = 45 (1) *n* = 22 (4.5% female) *M* = 62.5 ± 10.7 (2) *n* = 23 (8.7% female) *M* = 63.5 ± 8.1	5 months (1) progressively autonomous PA group: 2.5 months 2 supervised sessions and 1 autonomous session per week, 2.5 months 1 supervised session and 2 autonomous sessions per week, autonomy-supportive coaching style, individualized exercise prescriptions, pamphlet on PA, calendar (Gardner et al., 2012), 15 min phone interview every 2 weeks, SMS cues every day before PA session (2) supervised PA group: 2 supervised sessions and 1 autonomous session per week	Habit: SRBAI PA: IPAQ	Mixed model: Differences between groups in PA after the program n.s. General linear model: - Habit increases over time in both groups (η^2^ = 0.24, *p* = 0.012) - Differences between groups in habit n.s.
Kaushal et al. (2017a)	Dual-process approach (Evans, 2008), Habit model (Lally and Gardner, 2013)	Longitudinal observational design Baseline (2x SRBAI, GLTEQ) follow-up (2x SRBAI, GLTEQ, 6 weeks) Bivariate correlation, Structural equation models	Gym members *N* = 181 (64% female) *M* = 43.3 ± 15.3	No treatment	Habit: 2x SRBAI (specified for preparation and performance habit) PA: GLTEQ	Bivariate correlation between exercise at week 6 and preparatory habit (*r* = 0.22, *p* < 0.01) and performance habit (*r* = 0.20, *p* = 0.01) Structural equation models: - Habit preparation (ß = 0.20, *p* = 0.03) sig. predicts behavior at week 6 - Habit preparation (ß = 0.18, *p* = 0.04) predicts change in behavior from baseline to week 6
Kaushal et al. (2018) Canada	M-PAC	RCT Baseline (2x SRBAI, GLTEQ, accelerometer), post-test (2x SRBAI, GLTEQ, accelerometer, 8 weeks) Bivariate correlation, Mediation analysis	New gym members *N* = 94 (1) *n* = 41 (83% female) *M* = 38.21 ± 13.98 (2) *n* = 53 (81% female) *M* = 40.30 ± 14.69	8 weeks (1) intervention: workshop, consistent exercise plan, develop a preparatory exercise habit, implement cue rituals, booster telephone call (week 4) (for details see Kaushal et al., 2017b) (2) control: no treatment	Habit: 2x SRBAI (specified for preparation and performance habit) MVPA: GLTEQ, accelerometer	Bivariate correlations between - Habit preparation and self-reported MVPA (*r = 0*.25, *p < 0*.01) - Habit performance and self-reported MVPA (*r* = 0.23, *p* < 0.05) and accelerometry (*r* = 0.20, *p* < 0.05) Mediation analysis: - Preparatory habit (ß = 0.20, *p* = 0.04) predicts behavior change in self-reported PA - Indirect effect from group to self-reported PA via preparatory habit [ß = 0.10, CI (0.01, 0.33)]
Mullan et al. (2016) Australia		Longitudinal observational design Baseline (SRHI) t1 (PA 2 items, 1 week) Bivariate correlation, Hierarchical regression analysis	Pregnant women *N* = 117 *M* = 30.17 ± 4.46	No treatment	Habit: SRHI PA: 2 items “In the previous week, to what extent did you perform physical activity following the recommended guidelines?” “In the previous week, how often did you perform physical activity following the recommended guidelines?”	Bivariate correlation between habit and PA (*r =* 0.616, *p* < 0.001) Regression analysis: - Habit predicts PA (β = 0.33, *p* < 0.01) (model with gestation week, intention, PBC) - Interaction effect between intention and habit predicts PA (β = 0.31, *p* < 0.01) (model with gestation week, intention, PBC, habit)
Pfeffer and Strobach (2018) Germany	Strength model of self-control	Longitudinal observational design Baseline (SRBAI, PA 1 item), t1 (PA 1 item, 1 week) Bivariate correlation,Multiple regression analysis	University students *N* = 124 (52.4% female) *M* = 23.59 ± 2.76	No treatment	Habit: SRBAI PA: 1 item (number of hours of vigorous PA during last 7 days)	Bivariate correlation between: - Habit and PA at baseline PA (*r =* 0.41, *p* < 0.01) - Habit and PA at t1 (*r =* 0.29, *p* < 0.01) Regression analysis: - Habit (as moderator) predicts PA (ß = 0.23, *p* < 0.01) (model with age, sex, self-control) - Interaction effect between self-control and habit (as moderator) predicts PA (ß = 0.19, *p* = 0.028) (model with age, sex, self-control, habit) - Habit (as mediator) predicts PA at t1 (β = 0.28, *p* = 0.01) (model with age, sex, self-control)
Phillips and Gardner (2016) USA		Longitudinal observational design Baseline (2x SRBAI, PA 1 item) t1 (2x SRBAI, PA 1 item, electronic daily diary reports, 1 month) Bivariate correlation, Multiple regression analysis, ANOVA	University students and staff *N* = 118 Students (65% female) *M* = 19.48 ± 2.08 Staff/faculty (89% female) *M* = 37.61 ± 13.82	No treatment	Habit: 2x SRBAI (specified for preparation and performance habit) PA: 1 item “How often do you exercise?”, electronic daily diary reports	Bivariate correlation between instigation and execution habit and both PA outcomes at baseline and t1 (*r*-values between *r =* 0.22 and *r =* 0.56 with *p* < 0.05 and *p* < 0.01) Multiple regression analysis: only instigation habit predicts PA at t2 (daily diary report: β = 0.31, *t*_(3, 108)_ = 2.56, *p* = 0.01; PA 1 item: β = 0.39, *t*_(3, 107)_ = 3.16, *p* < 0.01) ANOVA: - Change in PA (from high at baseline to low at t1) associated with change in instigation habit (slope = −0.46, *p* < 0.05) - Change in PA (from low at baseline to high at t1) not associated with change in instigation habit (slope = 0.21, *p* = 0.22)
Rhodes et al. (2019b) Canada	TPB, M-PAC	Longitudinal two-arm intervention design Baseline (SRBAI, accelerometry) t1-t3 (accelerometry, mGLTEQ, weekly) t1 (SRBAI, 6 weeks) t2 (SRBAI, 13 weeks) t3 (SRBAI, 26 weeks) Bivariate correlation, Logistic regression analysis	Parents *N* = 102 (1) *n* = 50 (83% female) *M* = 42.96 ± 5.71 (2) *n* = 52 (75,5% female) *M* = 42.17 ± 5.68	26 weeks (1) education group: receiving information about the benefits of MVPA (2) education + planning group: receiving additional information on planning for PA for their child (for details see Rhodes et al., 2019a)	Habit: SRBAI PA: accelerometry, mGLTEQ	Bivariate correlation between habit and - PA via accelerometry at baseline (*r =* 0.20, *p* < 0.05) and at t1 (*r =* 0.33, *p* < 0.05), at t2 n.s. - PA via mGLTEQ across the trail (*r =* 0.36–0.51, *p* < 0.05) Regression analysis: - Habit predicts PA accelerometry from t1 to t2 of successful compared to unsuccessful intenders [OR = 1.99, *p* < 0.01; 95% CI (1.31, 3.04)] - Habit predicts mGLTEQ at all time periods [baseline to t1: OR = 1.60, *p* < 0.05; 95% CI (1.18, 2.17); t1 to t2: OR = 2.08, *p* < 0.01; 95% CI (1.33, 3.26); t2 to t3: OR = 1.88, *p* < 0.01; 95% CI (1.20, 2.93)]
Schwarzer et al. (2017) Italy, Spain, Greece	HAPA	RCT Baseline (SRBAI, GPPAQ) t1 (SRBAI, GPPAQ, 3 months) t2 (SRBAI, GPPAQ, 6 months) Bivariate correlation, Structural equation model	Adults *N* = 638 (1) *n* = 315 (61.9% female) (2) *n* = 323 (57.6% female) *M* = 43.01 ± 10.82	6 months (1) Intervention: dynamic digital platform (personalized paths to self-set goals, personalized feedback and rewards, weekly schedule according goals, strategies to overcome barriers) (2) Control: static digital platform (non-personalized information and feedback, recommendations according to baseline profile)	Habit: SRBAI PA: GPPAQ	Bivariate correlations between habit at t2 and - PA at baseline (*r =* 0.18, *p* < 0.01) - PA at t1 (*r =* 0.16, *p* < 0.01) - PA at t2 (*r =* 0.16, *p* < 0.01) Structural equation model: PA at t2 was predicted by habit at t2 (men: β = 0.17, women: β = 0.23) (model with motivation, planning at t1 and self-monitoring at t1)
van Bree et al. (2016) Netherlands	HAPA	Longitudinal observational design Baseline (intention, SRBAI, habit items, PA 1 item) t1 (action planning, 3 months) t2 (PA 1 item, 6 months) t3 (SRBAI, habit items, 12 months) Bivariate correlation, Regression analysis	Older adults **Study 1:** *N* = 469 (53% female) *M* = 63.07 ± 7.61 (data from an RCT-control group, for details see van Stralen et al., 2011) **Study 2:** *N* = 322 (49% female) *M* = 64.31 ± 9.39 (data from an RCT-control group, for details see Peels et al., 2013)	No treatment	**Study 1:** Habit: SRBAI PA: 1 item from SQUASH “On how many days per week are you, in total, at least moderately physically active for at least 30 min by undertaking, for example, heavy walking, cycling, chores, gardening, sports or other moderate or vigorous physical activities?” **Study 2:** Habit: 2 items from SRBAI and 2 items from SRHI: “Being sufficiently physically active is something … I do automatically, … I start doing before I realize I'm doing it, … I would find hard not to do, … I have no need to think about doing.” PA: like in study 1	**Study 1:** Bivariate correlation between - Habit (baseline) and PA (t2) (*r =* 0.18, *p* < 0.01) - PA (t2) and habit (t3) (*r =* 0.29, *p* < 0.01) Regression analysis: - PA (t2) predicts habit (t3) (â*b_*cs*_* = 0.18, *p* < 0.01) (model with intention at baseline and action planning at t1) **Study 2:** Bivariate correlation between - Habit (baseline) and PA (t2) (*r =* 0.37, *p* < 0.01) - PA (t2) and habit (t3) (*r =* 0.42, *p* < 0.01) Regression analysis: - PA (t2) predicts habit (t3) (â*b_*cs*_* = 0.21, *p* < 0.01) (model with intention at baseline and action planning at t1)
van Bree et al. (2017) Netherlands	Habit formation theory (Lally et al., 2008)	Longitudinal observational design Baseline (SRBAI, habit items, PA 1 item) t1 (SRBAI, habit items, PA 1 item, 6 months), t2 (SRBAI, habit items, PA 1 item, 12 months) Bivariate correlation, Structural equation models (cross-lagged panel design)	Older adults **Study 1:** *N* = 1.976* (57% female) *M* = 63.36 ± 8.66 (for details see van Stralen et al., 2011) **Study 2:** *N* = 2.140* *M* = 62.75 ± 8.57 (for details see Peels et al., 2013)	No treatment	**Study 1:** Habit: SRBAI PA: 1 item from SQUASH “On how many days per week are you, in total, at least moderately physically active for at least 30 min by undertaking, for example, heavy walking, cycling, chores, gardening, sports or other moderate or vigorous physical activities?” **Study 2:** Habit: 2 items from SRBAI and 2 items from SRHI: “Being sufficiently physically active is something … I do automatically, … I start doing before I realize I'm doing it, … I would find hard not to do, … I have no need to think about doing.” PA: like in study 1	Bivariate correlation between habit (baseline, t1, t2) and PA (baseline, t1, t2) in - Study 1 (*r =* 0.26–0.34, *p* < 0.01) - Study 2 (*r =* 0.38–0.51, *p* < 0.01) Structural equation models: **Study 1:** - Mediation effect for habit(baseline) -PA(t1)-habit(t2) path [product of coefficients' *z* = 2.73, *p* < 0.01, CI (0.004; 0.019), â*b_*cs*_* = 0.011 *PME* = 2.7%] - Marginal sig. for PA(baseline)-habit(t1)-PA(t2) path [*z* = 1.84, *p* = 0.067, CI (0.000; 0.011), â*b_*cs*_* = 0.01, *PME* = 2.4%] **Study 2:** - Mediation effect for the path PA(t0)-habit(t1)-PA(t2) [*z* = 4.07, *p* < 0.01, CI (0.016; 0.044), â*b_*cs*_* = 0.03, *PME* = 10.8%] - Mediation effect of the habit(t0)PA(t1)-habit(t2) path n.s.
White I. et al. (2017) UK	Habit-formation model (Gardner et al., 2012; Lally and Gardner, 2013)	RCT Baseline (SRHI 1 item, IPAQ) t1 (SRHI 1 item, IPAQ, 8 weeks) t2 (SRHI 1 item, IPAQ, 12 weeks) Bivariate correlation	Older adults *N* = 90 (1) *n* = 45 (60% female*) *M* = 68 ± 4.05* (2) *n* = 45 (57% female*) *M* = 68.61 ± 3.52*	12 weeks (1) Intervention: information booklet outlining the health impact of SB and PA, 15 tips on reducing SB and forming PA habits, tick-sheets to record daily adherence to tips (2) Control: factsheet outlining the health consequences of PA and SB, UK recommendations for duration, frequency and intensity of PA, activity examples to increase PA and reduce SB	Habit: SRHI 1 item (PA is something I do without thinking) PA: IPAQ (walking, moderate, vigorous)	Bivariate correlations between habit and PA for (1) and (2) n.s.

* *Only reported for baseline*.

*PA, physical activity; TPB, Theory of Planned Behavior; SRHI, Self-Report Habit Index; n, sample size; M, mean age; HAPA, Health Action Process Approach; RIM, Reflective Impulsive Model; SRBAI, Self-Report Behavioral Automaticity Index; eTPB, Extended Theory of Planned Behavior; IPAQ, International Physical Activity Questionnaire; min, minutes; RRR, relative risk ratio; SMS, short message service; n.s., not significant; ANOVA, Analysis of Variance; GLTEQ, Godin Leisure Time Exercise Questionnaire; M-PAC, Multi-Process Action Control; RCT, Randomized-controlled Trail; MVPA, Moderate to Vigorous Physical Activity; CI, Confidence Interval; mGLTEQ, modified Godin Leisure-Time Questionnaire; OR, Odds Ratio; GPPAQ, General Practice Physical Activity Questionnaire; SQUASH, Self-administered Dutch short questionnaire to assess health-enhancing; SB, sitting behavior*.

## Discussion

The purpose of this review was to investigate the relationship between habit and physical activity behavior in longitudinal studies. Our first hypothesis did not focus on the longitudinal study design, but nevertheless the majority of studies supported that positive correlations between habit and physical activity exist. Furthermore, our second hypothesis was that higher habit strength leads to an increased level of physical activity and vice versa. Positive direct effects of habit on physical activity were detected in only two studies. Several studies presented indirect effect sizes, which cannot be compared to each other due to the influence of several psychological variables on the relationship of habit and physical activity in different model constellations. Therefore, a confident confirmation for this direction of effect is not possible. Only one study revealed that physical activity influenced habit strength and two other studies showed the effect of physical activity interventions to strengthen habit. Because of the insufficient number of presented studies, the hypothesis cannot be confirmed satisfactorily. One study applied a cross-lagged panel design to answer both directions of effect and reported inconclusive results. A distinction between instigation or preparatory and execution or performance habit was conducted in three studies, which showed positive correlations for both habit variables and physical activity. These studies supported the third hypothesis, namely that the relationship between instigation or preparatory habit and physical activity is stronger than that between execution or performance habit and physical activity.

### Theoretical Background Considerations

Studies based on cognitive theories added habit as a non-conscious construct to explain physical activity behavior. Similar to dual-process approaches (Evans, 2008; Evans and Stanovich, 2013; Presseau et al., 2014), both conscious and non-conscious processes are relevant to force behavior change. Therefore, it is advisable to combine both approaches as a holistic theoretical foundation. To realize this in future research, the framework M-PAC by Rhodes (2017) could be helpful for intervention studies to focus on habit formation and conscious processes (Kaushal et al., 2018; Rhodes et al., 2019b). The framework involves different phases of behavior, from initiation to continuation, and recommends appropriate targets, such as triggering cues (Rhodes, 2017). Another approach was recently introduced by Strobach et al. (2020). This heuristic Physical Activity Adoption and Maintenance (PAAM) model includes both implicit and explicit processes as the basis for physical activity maintenance (Strobach et al., 2020).

Beyond that, a consistent habit theory should be considered. There are various definitions of habit existing from different authors. Instead of revising existing theories of habit, scientists should take into account a common consensus about central elements characterizing habit. Although Lally and Gardner (2013) already developed a framework for habit formation, only two studies of this review used this as theoretical background to develop new habits (Kaushal et al., 2017a; White I. et al., 2017). In their paper, Lally and Gardner (2013) broke the habit formation process down into detailed stages which can be applied in interventions to develop a physical activity-related habit. Future research could be more efficient with a unitary understanding of the habit construct and, thus, tap the full potential of habit and its effects on physical activity behavior.

### Contribution to Habit Research

The positive correlations between habit and physical activity indicates that habit plays an important role in physical activity promotion. However, the question remains whether habit influences physical activity or vice versa. Only a few studies analyzed the direct effect of habit on physical activity, but this helps to solely quantify the relationship. Most included studies used complex models with other psychological variables and could only present indirect effects of habit. Due to the heterogeneity of study designs, conclusions about the effect of habit on physical activity must be treated with caution. However, included studies can contribute to topics currently discussed in habit research.

Intention was a central variable in various studies and different approaches were used to relate the constructs of intention and habit to explain behavior. This reflects the relevance of the current discussion about the complex interplay between intention, habit, and behavior, which needs to be clarified. In a recently published review, Gardner et al. (2020b) addressed the habit-intention interaction hypothesis of Triandis (1977) and examined 52 studies focusing on the predictive effect of the habit-intention relationship on behavior. Physical activity studies showed mixed support of the hypothesis that habit impulses will preside over intentions in stable settings. In conclusion, the authors argued that habits have the potential to dominate over intentions and intentions have the potential to dominate over habits, but this interaction depends on the level of self-control. Our review does not support the assumption of previous research that intention becomes less predictive of physical activity as habit strength increases. In several studies, no direct relationship between intention and habit was found, which significantly contributed to explain physical activity (Allom et al., 2016; Mullan et al., 2016; van Bree et al., 2016; Kaushal et al., 2017a). Authors concluded that intention and habit should be viewed as individual variables influencing physical activity behavior on different paths. Conscious and non-conscious processes both independently contribute to behavior change, which supports the idea of a dual-process approach (Allom et al., 2016; Mullan et al., 2016; Kaushal et al., 2017a). Therefore, we consider intention as a key variable for physical activity behavior, besides individual habit strength. We agree with Kaushal et al. (2017a) that both variables are highly relevant in physical activity behavior change processes and should not compete against each other.

Some authors focused on self-regulatory processes such as self-control (Pfeffer and Strobach, 2018), which was operationalized through planning and self-monitoring in intervention studies (Schwarzer et al., 2017; White I. et al., 2017; Fournier et al., 2018; Kaushal et al., 2018; Rhodes et al., 2019b). Self-regulatory strategies might have an essential role in the habit formation process. Also, Lally and Gardner (2013) incorporated these strategies in their framework and recommended both planning and self-monitoring for action initiation and behavior repetition, resulting in successful habit formation. Only a few studies applied self-regulatory constructs and presented inconclusive results on the effect of planning and self-monitoring on habit formation (Schwarzer et al., 2017; White I. et al., 2017; Fournier et al., 2018; Kaushal et al., 2018; Rhodes et al., 2019b). Nevertheless, authors proposed planning and self-monitoring as relevant variables, which could support the development of habits as Lally and Gardner (2013) already stated. Especially planning is conducive to the habit formation process by acting as a cue-related factor and “thus provides the cognitive architecture through which intentional actions may become habitual” (Gardner and Lally, 2018, p. 213).

When discussing the effect of physical activity on habit, Gardner (2015) distinguished between habit formation studies and studies measuring ongoing habits, because only formation studies have the potential to create new habits. Two studies aimed to build habits and successfully used physical activity as intervention content to form habit (Fournier et al., 2016, 2018). These results show that a physical activity program itself can effectively contribute to develop habits. This concurs with the framework by Gardner and Lally (2018), in which behavior repetition is an important step of the habit formation process. One observational study found an indirect effect, in which most likely an ongoing habit was predicted via physical activity frequency (van Bree et al., 2016). However, the causality of a behavior-habit effect remains unclear. The prediction of an ongoing habit through physical activity could result in false conclusions, because the existing habit positively influenced physical activity beforehand. Therefore, this explains the prediction of physical activity through habit rather than the prediction of habit through physical activity.

Phillips and Gardner (2016) were the first authors to distinguish between instigation and execution habit. To our knowledge this is the only experimental study investigating the role of these constructs in physical activity behavior so far. In this study instigation habit was a significant predictor of physical activity. The authors assumed that the habitual instigation of physical activity might be more valuable for a regular performance of behavior than the habitual execution of the behavior itself. Execution habit was not found as a predictor in their study. However, Gardner et al. (2020a) hypothesized that the habitual execution of physical activity may influence other constructs such as self-efficacy or affective judgments resulting in a higher physical activity engagement. The potential of execution habit has not been evaluated in experimental studies yet. The hypothesis of Gardner et al. (2020a) should be examined in future research to investigate the role of execution habit during physical activity sessions. Furthermore, we assume that the quality and volume of training could be ensured through the habituation of certain exercise sequences. Especially in intervention studies the content and performance of physical activity could be warranted through execution habits. It may be more successful to focus not only on the initiation of physical activity but also on the output of physical activity sessions to fulfill the international physical activity recommendations.

Different to the approach of Phillips and Gardner (2016), the distinction between preparatory and performance phase could be a practical concept in habit research. In the studies of Kaushal et al. (2017a, 2018), the assessment of habit refers to the behavioral phases before and during the exercise behavior. The preparatory phase of exercising consists of several sequences of preparatory actions like packing the gym bag or going to the gym. In the view of Phillips and Gardner (2016), these actions belong to the execution of the whole exercise behavior. However, they do not distinguish in the assessment of habit between habitually doing before and during the exercises. The assessment of execution habit by Phillips and Gardner (2016) focused only on cue-behavior associations while exercising (“Once I am exercising… [… is something I do automatically]”). Therefore, habitual preparatory actions were only assessed in the studies of Kaushal et al. (2017a, 2018) and findings revealed preparatory habit as the significant predictor for exercising. Altogether, the approaches of Phillips and Gardner (2016) and Kaushal et al. (2017a, 2018) both have the eligibility to contribute to habit research, but it may be that the combination of these concepts encapsulates the habit-behavior relationship in its entirety. Consequently, the assessment of habitually deciding and habitually doing, which could be divided into a preparatory and performance phase, would result in three SRHI-stems: “Deciding to exercise… [… is something I do automatically],” “When I prepare to exercise… [… is something I do automatically],” “Once I am exercising… [… is something I do automatically].” Future studies could examine and compare the predictive power of these SRHI-stems to gather more information about the relevance of the approaches of Phillips and Gardner (2016) and Kaushal et al. (2017a, 2018) in the up-take or maintenance of physical activity.

### Conceptual Considerations

This review focused on studies applying the SRHI or SRBAI to assess habit. Previously published literature showed that these instruments are the most commonly used habit measures (Lally and Gardner, 2013; Gardner, 2015; Rebar et al., 2016). Based on homogeneity, changes in habit strength are comparable between studies (Rebar et al., 2016). However, data relies on self-report, resulting in questionable construct validity, because non-conscious regulatory processes may not be precisely expressed (Gardner and Tang, 2014; Rebar et al., 2016). Using self-reported measurements to assess unconscious processes might be a general problem in examining implicit constructs. Because of its automatic character, habit is performed without deliberating behavioral choices.

A reliable measure of habit should incorporate all components of habit, especially contextual cue dependency as a central characteristic of habit, which is neglected in the SRHI and its variations such as the SRBAI (Gardner, 2015). The original survey should be adapted, involving the contextual cue related to the behavior (“Behavior X in Context Y is something I do automatically.”) (Gardner, 2015, p. 283; Hagger, 2019). Rebar et al. (2016) recommended alternative methods not relying on self-reported measures to ensure validity of the habit assessment. Theoretically, implicit methods would be the most valid measures, but the practicability in experimental field studies is insufficient (Gardner, 2015). Therefore, self-reported measures are still the dominating assessment tool in applied research. One option to measure habit in daily life is to apply Ecological Momentary Assessment (EMA) methods, which could show that habitual responses depend on environmental contexts. Digital technologies such as smartphones and tablets can record large amounts of data and this enables a detailed analysis of habits in everyday life (Carden and Wood, 2018). EMA methods offer the opportunity to measure behavior in real-time and allow researchers to assess various factors simultaneously. Psychological components can be assessed directly before, during, or after physical activity, reflecting reality more accurately. Moreover, objective and subjective measurements, such as accelerometer and self-reported questionnaires, can be combined in experimental studies. Additionally, the application of wearable technology could facilitate the efficient implementation of complex study designs (Ebner-Priemer et al., 2019).

The application of EMA methods would contribute to the demand of Lally and Gardner (2013) for multiple measurements over long time periods to assess habit. Several measurement occasions would make the habit formation process related to physical activity behavior more comprehensible and could show changes in habit and physical activity behavior. To realize effects over time and relate habit to physical activity, the assessment of both constructs at all measurement occasions is required. Although the amount of measurement occasions varied highly in the included studies, several authors presented promising approaches to fulfill these recommendations (Schwarzer et al., 2017; van Bree et al., 2017; White I. et al., 2017; Fournier et al., 2018; Rhodes et al., 2019b). So far, longitudinal studies are rare, because high-quality studies over a long time period are costly and time consuming. The included study by Rhodes et al. (2019b) could serve as an example for the application of state-of-the-art technology to assess physical activity behavior and for the implementation of multiple measurement occasions for habit. Physical activity behavior was tracked with an accelerometer in everyday life for 6 months and, in addition, habit data were collected via self-report on four occasions within this period. This study design met central recommendations published by Gardner and Lally (2018). Three other studies measured habit and physical activity only once at different measurement occasions and related the two constructs (Allom et al., 2016; Arnautovska et al., 2016; Mullan et al., 2016). Even though this study design has been considered as longitudinal, the development of both habit and physical activity cannot be analyzed. Therefore, this study design is not advisable for measuring long-term effects. In future research, study designs with many measurement occasions are required.

When discussing the study design, also study analysis methods should be reconsidered. The construct of habit depends on cue, behavior, and person-related factors. Hence, sum scores for habit across all participants are insufficient because the habit-behavior relationship develops individually. Consequently, within-designs in multi-level analysis with multiple measurement occasions would be most valuable (Gardner and Lally, 2018). Two studies applied mixed-model analysis to capture the influence of the intervention program on the participants' habit and physical activity behavior (Fournier et al., 2016, 2018). When focusing on the direction of causality between the two constructs, cross-lagged panel designs could be beneficial (Pfeffer and Strobach, 2018). This design was adopted by van Bree et al. (2017) to model a longitudinal relationship between the constructs in three waves in two different study populations. This allowed a simultaneous analysis in both directions. Due to contrary findings in both studies, the question of causality remains unanswered. However, further studies applying this study design are needed to investigate the bidirectional association of habit and physical activity.

### Habit Formation and Physical Activity Maintenance

Only a few included studies aimed to form new habits through intervention designs (Fournier et al., 2016, 2018; Schwarzer et al., 2017; White I. et al., 2017; Kaushal et al., 2018; Rhodes et al., 2019b). A central question in the literature is how long it takes to develop a certain habit. Even though these habit formation studies revealed positive effects on the habit-behavior relationship, the habit development process was transparent in only one study (Fournier et al., 2016). Fournier et al. (2016) measured the weekly strength of habit and could show the development over 28 weeks. SRBAI-scores increased over the intervention period stepwise. One plateau was reached between week 6 and 8, which is in line with previous studies. To our knowledge, only three other studies examined the time needed for habit formation. Lally et al. (2010) found that the development of a habit is asymptotic, which means there is a point from which the growth of habit strength is only marginal. A median of 66 days was calculated to reach 95% of the asymptote. Contrary to that, in the study by Fournier et al. (2016), the increase of habit strength was distributed over the complete intervention period of 28 weeks with the highest scores in the last few weeks. In another previous study, Kaushal and Rhodes (2015) showed that habit formation was reached after 6 weeks, with participants exercising four times each week. A total of 48% of the study population had formed a habit after 42–49 days of intervention. Walter (2017) presented similar results and detected a stagnation of SRHI scores after 8 weeks. Unfortunately, the latest intervention studies cannot contribute to the question of how long it takes to form a habit, because descriptive data in the form of means are not enough to observe that. However, intervention periods of at least 6–8 weeks are advisable, but long-term study designs should be applied to give confident recommendations for future research.

The habit formation process depends on how quickly an individual experiences a behavior as habitual (Hagger, 2019). For the identification of an action as habitual, cues in both habit and behavior must be specified (Gardner, 2015). Additionally, Hagger (2019) stated that habit formation takes not only time, but also the presence of key factors, like intention strength, perceived behavioral complexity, and the use of self-regulatory skills, is necessary. Many of the included studies involved some of these factors as variables in their models, but as discussed before, the individual development of habit was not taken into account. This procedure can be found in the study by Schwarzer et al. (2017) where motivation, planning, self-monitoring, habit strength, and physical activity were placed in a sequential manner and each variable assessed only once. Hence, the development of habit strength was not documented. Therefore, a possible influence of key factors on habit formation was not detectable. Schwarzer et al. (2017) viewed habit strength as the proximal predictor of physical activity and assumed that planning and self-monitoring become less predictive for physical activity behavior when habit strength increases. Future habit formation studies should therefore not only extend the length of intervention in combination with frequent measurement occasions, but also focus on individual differences and key factors determining habit formation. Besides, there is no threshold within the SRHI to ensure whether a habit has already been formed or not. Focusing on the influence of key factors on habit formation seems only beneficial when habit strength in participants is initially weak. Then, habit strength can develop, and one requires continuous assessment of habit over time.

Long-term studies are not only relevant for the development of habit, but also for stabilizing a new health behavior like physical activity. The overall goal is to maintain physical activity behavior after interventions as a part of the participants' daily lives. The repetition of physical activity over a longer period of time can be understood as a pattern of behavior called physical activity maintenance (Kahlert, 2015). The duration needed to reach physical activity maintenance is still unclear due to a missing consent on cut-off values (Kwasnicka et al., 2016). Kwasnicka et al. (2016) underlined in a systematic review that habit is the most continuous variable for physical activity maintenance, especially when self-regulation is low and only minimum levels of awareness and resources are available. Last measurement occasions in long-term studies included in this review were conducted between 6 months to 2 years (Bird et al., 2018) post-baseline measurement. Fournier et al. (2016) measured only physical activity after 19 months. Other authors measured both habit and physical activity at follow-up (van Bree et al., 2017; Bird et al., 2018; Fournier et al., 2018; Rhodes et al., 2019b). Contrary to long-term study designs, few observational studies narrowed their duration down to 1 week, measuring the effect of baseline habit on physical activity after 1 week (Allom et al., 2016; Arnautovska et al., 2016; Mullan et al., 2016; Pfeffer and Strobach, 2018). This does not reflect the effect of habit as a sustainable variable on physical activity maintenance. Furthermore, it is questionable whether long intervals, such as 12 months, between the measurement of habit and physical activity are effective, because it is unlikely that habit predicts physical activity 1 year later without showing a development of both variables over time (Bird et al., 2018). However, it is still unclear how long it takes to reach physical activity maintenance and therefore, long-term study designs with regular measurement occasions are deemed necessary in future habit research.

### Study Quality

The applied quality assessment tool showed that study quality was equally poor, fair, and good across 15 studies. Overall, study quality of the included studies was reasonable. As mentioned before, some studies neglected the repeated measurement of both variables habit and physical activity (Allom et al., 2016; Arnautovska et al., 2016; Mullan et al., 2016), which was one reason why study quality was rated as poor. Another critical factor for poor quality was missing power analyses before the implementation of the study procedure (Allom et al., 2016; Arnautovska et al., 2016; Mullan et al., 2016; Bird et al., 2018). According to the quality assessment tools of the NHLBI (NHLBI, 2014), studies with poor quality show a significant risk of bias. Therefore, these study results must be treated with caution. A limitation of the applied assessment tools is that the overall rating relies on the reviewers' perception and not on a defined ranking.

Although two of the five studies being rated with poor quality, we would like to highlight some positive aspects. Besides other limitations, Bird et al. (2018) did not perform a power analysis, but presented a much higher sample size compared to other studies. Additionally, the study was conducted under real-world conditions which exacerbates the data collection on more time occasions, but increases the external validity of the study. Also, the study quality of Kaushal et al. (2018) was rated poorly due to methodological weaknesses. They did not address whether the participants were blinded in the RCT, and used data of participants not attending the intervention workshop for analysis in the control group. However, the detailed description of the intervention content gained our attention. Kaushal et al. (2018) applied various behavior change techniques derived from Michie et al. (2013). In terms of habit formation, they focused on repetition and substitution with the goal to form a new habit. Additionally, the M-PAC framework was tested for the first time in an RCT and this could be a promising approach for habit formation studies.

## Limitations

The systematic search was carried out in only three databases, which is why a thorough snowball search was performed. The only inclusion criteria concerning study design was a longitudinal approach, and no further restrictions were added. Included studies focused on different research questions and this resulted in a high heterogeneity of study designs. Only studies measuring habit with the SRHI or a form of this measurement were included. As discussed before, the SRHI is widely used to capture habit, but the validity is questionable due to self-reported data. Measurement tools for physical activity were diverse, hence a comparability of physical activity content and frequency is not possible. Only a few studies analyzed the direct effect of habit on physical activity, and vice versa, and could therefore answer our hypothesis. For this reason, the findings of this review are limited.

## Conclusion and Future Directions

This review revealed a bidirectional relationship between habit and physical activity. Whether habit predicts physical activity or vice versa is still unclear. Based on the findings of included studies, we conclude that the direction of prediction analyses between habit and physical activity depends on the objective of research. The observation of habit influencing physical activity may be most appropriate in studies fostering physical activity maintenance, while the influence of physical activity on habit may be reasonable in experimental studies with physical activity as intervention content to form a habit. In habit research, we advise the distinction between instigation and execution habit, because both variables support the habit formation process independently.

In addition to the recommendations based on the evidence we gathered through the conduction of this systematic review, approaches of habit formation in health behaviors other than physical activity should be considered for future research as well. For example, Fritz et al. (2019) showed in a feasibility study for a weight loss intervention that African-American participants were successful in improving their nutrition and physical activity behavior through the self-selection of new habits they wanted to adopt. Typically, the health behavior for which a habit should be developed is determined by the researchers before the study begins. Especially with the aim to develop new habits, it may be an important precondition in terms of intrinsic motivation to let participants self-select appropriate habits to facilitate nutrition and physical activity behavior. This approach was also applied successfully in the physical activity domain by Lally et al. (2010). Furthermore, weight loss interventions integrated the environment of participants as a central component influencing the habit formation process (Carels et al., 2014; Fritz et al., 2019). Therefore, the modification of the environment could enhance habit strength through triggering cues which are positively related to the target behavior. Also, Bird et al. (2018) applied this approach through an infrastructure project to increase walking and cycling. On the one hand, communities and researchers can create environmental conditions to promote habit formation. On the other hand, it seems beneficial to enable participants to construct their individual environment on their own with the aim to establish healthy habits. Regarding the intervention content, weight loss interventions (Carels et al., 2014; Cleo et al., 2019) distinguish between the formation of a “good” habit and the disruption of an existing “bad” habit (Gardner and Rebar, 2019). Studies showed inconsistent results on whether the disruption of a “bad” habit solely promotes health behavior (Cleo et al., 2019) or whether a combination including the formation of a “good” habit is more promising (Carels et al., 2014). Only one study in this systematic review addressed sitting behavior as an unhealthy habit and tried to displace it with a habit for physical activity behavior (White I. et al., 2017). Although findings of this pilot study were not significant due to methodological limitations, the authors concluded that a habit-based intervention with the target to replace a “bad” with a “good” habit is promising, but that it needs further development. Future intervention studies focusing on habit formation should involve many measurement occasions (e.g., weekly) and should last at least 6–8 weeks. The framework of habit formation may be a helpful basis to conduct a promising study design including key factors for habit development, such as planning and self-monitoring. Complex studies involving variables that may influence the habit development process or disturb the maintenance of habitual behavior could be beneficial. For example, Weyland et al. (2020) investigated in a longitudinal study the influence of affect in habit formation. They showed that positive affect was significantly related to automaticity while repetition was not. Based on these results, we recommend further investigations involving determining factors of habit, such as affect. Because the process of habit formation varies between persons, the application of within-designs in future investigations is necessary. In line with Rebar et al. (2016), future studies should focus on physical activity maintenance, so that habit can function as an automatic process variable, stabilizing physical activity behavior. Long-term studies should investigate the role of habit in different stages of physical activity behavior change and maintenance. The relationship of habit and physical activity could develop over time and should therefore be observed regularly.

## Data Availability Statement

The original contributions presented in the study are included in the article/Supplementary Material, further inquiries can be directed to the corresponding author/s.

## Author Contributions

KF, SA, and SW generated the search term for this topic under the supervision of DJ. The screening process was allocated equally among KF, SA, and SW. KF and SA conducted the data extraction of included studies and synthesized the results of this review together. Each study was assessed for quality by KF, SA, and SW independently. The discussion was mainly conducted and written by KF and assisted by SA. SW and DJ contributed to the conceptualization of the review and revised the manuscript prior to publication. All authors contributed to the article and approved the submitted version.

## Conflict of Interest

The authors declare that the research was conducted in the absence of any commercial or financial relationships that could be construed as a potential conflict of interest.
